# Diabesity management worldwide

**DOI:** 10.4155/fso.15.86

**Published:** 2016-03-01

**Authors:** Chinnadorai Rajeswaran

**Affiliations:** 1 Mid Yorkshire NHS Trust, UK

**Keywords:** diabesity, diabetes, GLP-1 analogues, obesity

## Abstract

**Francesca Lake, Managing Editor, speaks to Chinnadorai Rajeswaran.** Dr Rajeswaran is a consultant physician (Diabetes & Endocrinology) at Mid Yorkshire NHS Trust. He gained specialist training in Leeds, in diabetes and endocrinology. He has a special interest in obesity and is involved in research in obesity and diabetes. He also has a number of publications, book chapters and presentations to his credit. He along with other co-authors has published a book on weight loss surgery, titled: “The Ultimate Guide to Weight Loss Surgery.” He leads the specialist obesity service at the Mid Yorkshire Trust and Kirklees weight management service. With the help of colleagues in diabetes and obesity he set up the National Diabesity Forum. Dr Rajeswaran is also the medical advisor for Simplyweight, a global specialist weight management organization. He is also involved in both local and international charity work.

## Q Can you tell us a little about your career to date and what brought you into weight management and diabetes?

I have been a consultant for nearly 9 years and I have been trained in diabetes and endocrinology. However, over the past few years I have noticed that most of our patients with Type II diabetes, approximately 80%, have some degree of weight problem; they are overweight or obese. The problem is, improving glycemic control is very closely related to weight, and so it can be difficult to manage glycemic control and other issues common in individuals with Type II diabetes. That is why I moved into work in obesity, and now most of my work is in weight management.

## Q You founded the National Diabesity Forum, can you tell us a little about it?

The National Diabesity Forum is an educational forum to aid the dissemination of knowledge to healthcare professionals, including nurses, obesity nurses, dietitians, surgeons and physicians, for example, providing practical ways and guidance on how to manage individuals who have diabetes and obesity. We publish the journal *Diabesity in Practice* and we also run online programs, where we have lectures and also provide protocols and guidelines for the management of diabetes and obesity. We also update healthcare professionals about the latest research that is going on in diabetes and obesity.

## Q What led to you setting up the National Diabesity Forum?

There is no institution or organization that specifically deals with obese people with diabetes or diabetic people with obesity, so that was the reason behind setting up the forum. There is a huge cohort of people where it is not just the glucose, it is also the weight that is the problem. Both aspects have to be tackled, but because of limited resources, we need to ensure that diabesity management is incorporated into every diabetes clinic. The Forum was founded 7 years ago and is now well-known throughout the UK, with many subscribers.

## Q What other research are you working on at the moment?

I am currently supervising two PhD students; one is looking into the psychological aspects post-bariatric surgery and the psychology of individuals with weight issues and the second is investigating high-intensity interval training in obese individuals. The benefits of high-intensity interval training are well known in very fit people; however, not as much is known regarding its impact in obese people.

## Q How do you foresee these having an effect in the clinic?

Most of the problems with any chronic illness are related to psychology. Obesity is a chronic illness and we need to treat it as such, and continually support people with weight issues. Psychological issues are a key part of the reason why people regain weight after losing it. These are issues that are very rarely dealt with, and unless we understand the background of people with weight problems we will never rectify this issue. It is very expensive to provide psychological services for people with weight issues, therefore this research intends to design protocols for how individuals can be classified and graded so that the neediest patients are provided with input from a clinical psychologist. It is hoped that this could also improve cost–effectiveness and provide better services for patients.

## Q What do you find the greatest challenge in your field at the moment?

The greatest challenge in individuals with diabetes and obesity is engagement; in other specialties such as cardiology or oncology there is ‘hype’. For example, in cardiology, if a patient is experiencing chest pains they will seek immediate help. In oncology, the patient worries about the life-limiting effect of the cancer and will seek help immediately. Whereas in diabetes and obesity, as the patient is not in pain, individuals do not seek help and treat the condition as seriously as they should. Getting patients to engage with appropriate treatments for obesity and diabetes is a real challenge: how can we ensure they take treatment seriously and follow the doctor’s instructions?

The second most important challenge is: how do we provide a personalized service? We cannot provide a blanket management strategy as every person with diabetes and obesity has a unique underlying cause, so personalizing management is a challenge. Developing innovative models to address the psychological issues that patients present with is also a big challenge due to time constraints and lack of skilled healthcare professionals.

## Q How do you think we need to go about addressing these issues?

One main aspect is to carry out more research into the psychological framework of people with diabetes and obesity and try and classify people who are in most need of expert psychological assessment. A second aspect would be to provide a personalized service, for which we would require experts who could provide such a service, both offline and online. The provision of hospital-based services would be very expensive, as would community-based services, due to the fact that one-third of the UK population has some degree of either problem. In my opinion, an online service combined with an offline service would be the best option.

## Q NICE has recently released a second draft of their Type II Diabetes Guidelines [1]; can you tell us what is new in the guidelines?

The second draft has improved on the first; however, there are still some shortcomings that need to be addressed before the guidelines are finalized. The first aspect is the prescription of the recommended standard-release metformin. It needs to be recognized that patients who cannot tolerate standard-release metformin need to be prescribed slow-release metformin. The second line of drugs is sulfonylurea. That is new; it was originally just repaglinide, but now we have gliptins, sulfonylurea, pioglitazone, etc. What concerns me is the non inclusion of GLPs or SGLT as second line for the most appropriate patients. As I said, I am very keen on addressing weight, because if you do not act this way, people put on weight, and they would disengage from the clinic. Weight issues have to be addressed early; you cannot keep waiting and eventually introduce a treatment after they have gained weight. The newer group of drugs should be used appropriately and that would improve the cost–effectiveness of our diabetes care. So, I believe that GLPs and SGLTs should be placed more appropriately in the second draft. For people who are intolerant of metformin, nothing is mentioned in regards to GLPs or SGLT; there is currently no indication to use this class of drugs for people who are intolerant of metformin.

## Q So, you agree with the positioning statement released by AstraZeneca regarding the need for information regarding personalized care?

Absolutely, I feel that patients should benefit from the prescription of new medicines. I agree that medications are expensive and we need to be careful with the limited resources that we have. But, in delaying treatment for people in need, we are inadvertently increasing expenses in the long run. I believe in holistic management and treating appropriately and cost effectively early on. Ideally, someone would be prescribed metformin, and then sulfonylurea and pioglitazone. If blood glucose control is not reduced or weight gain is pronounced, patients may disengage and not be complaint with the management plan. If a GLP is considered at a later stage, it may not be effective. The GLP-1 class of drugs are effective only if there is adequate beta cell reserve, this clearly indicates that this class of drugs should be introduced early on, so as to be cost effective.

## Q Thank you. So in general, how would you like to see diabetes care changing over the next 10 years?

I think there will be further new medications developed, and most of these medications will be targeting weight. Bariatric surgery will become more popular within the management of diabetes and I believe this is the way forward.

## Q Finally, if you had unlimited resources, what would you make it your priority to do in this field?

I would prioritize more research into personalizing diabetes care. Trying to find out, even before we start prescribing medication, which medication is suitable for that particular individual. Developing methods to determine the pancreatic reserve before we start medication would be extremely useful, but for this we need adequate funding and motivated healthcare professionals.
